# Defining characteristics of genital health in South African adolescent girls and young women at high risk for HIV infection

**DOI:** 10.1371/journal.pone.0213975

**Published:** 2019-04-04

**Authors:** Smritee Dabee, Shaun L. Barnabas, Katie S. Lennard, Shameem Z. Jaumdally, Hoyam Gamieldien, Christina Balle, Anna-Ursula Happel, Brandon D. Murugan, Anna-Lise Williamson, Nonhlanhla Mkhize, Janan Dietrich, David A. Lewis, Francesca Chiodi, Thomas J. Hope, Robin Shattock, Glenda Gray, Linda-Gail Bekker, Heather B. Jaspan, Jo-Ann S. Passmore

**Affiliations:** 1 Institute of Infectious Disease and Molecular Medicine (IDM), University of Cape Town, Cape Town, South Africa; 2 DST-NRF CAPRISA Centre of Excellence in HIV Prevention, Durban, South Africa; 3 Desmond Tutu HIV Foundation (DTHF), University of Cape Town, Cape Town, South Africa; 4 SAMRC-UCT Gynaecological Cancer Research Center (GCRC), University of Cape Town, Cape Town, South Africa; 5 National Health Laboratory Service, Groote Schuur Hospital, Cape Town, South Africa; 6 Centre for HIV and STIs, National Institute for Communicable Disease (NICD), National Health Laboratory Service, Johannesburg, South Africa; 7 Perinatal HIV Research Unit (PHRU), Faculty of Health Sciences, University of the Witwatersrand, Diepkloof, Johannesburg, South Africa; 8 Western Sydney Sexual Health Centre, Western Sydney Local Health District, Parramatta, Australia; 9 Marie Bashir Institute for Infectious Diseases and Biosecurity & Sydney Medical School-Westmead, University of Sydney, Sydney, Australia; 10 Karolinska Institute, Stockholm, Sweden; 11 Northwestern University, Chicago, IL, United States of America; 12 Imperial College London, London, United Kingdom; 13 South African Medical Research Council, Cape Town, South Africa; 14 Seattle Children’s Research Institute, University of Washington, Seattle, Washington, United States of America; Instituut voor Tropische Geneeskunde, BELGIUM

## Abstract

The genital tract of African women has been shown to differ from what is currently accepted as ‘normal’, defined by a pH≤4.5 and lactobacilli-dominated microbiota. Adolescent girls and young women (AGYW) from sub-Saharan Africa are at high risk for HIV, and we hypothesized that specific biological factors are likely to be influential. This study aimed to compare characteristics of vaginal health in HIV-negative AGYW (16-22-years-old), from two South African communities, to international norms. We measured plasma hormones, vaginal pH, presence of BV (Nugent scoring), sexually transmitted infections (multiplex PCR for *Chlamydia trachomatis*, *Neisseria gonorrhoea*, *Trichomonas vaginalis*, *Mycoplasma genitalium*) and candidiasis (Gram stain) in AGYW (n = 298) from Cape Town and Soweto. Cervicovaginal microbiota was determined by 16S pyrosequencing; 44 genital cytokines were measured by Luminex; and cervical T-cell activation/proliferation (CCR5, HLA-DR, CD38, Ki67) was measured by multiparametric flow cytometry. 90/298 (30.2%) AGYW were negative for BV, candidiasis and bacterial STIs. *L*. *crispatus* and *L*. *iners* were the dominant bacteria in cervicovaginal swabs, and the median vaginal pH was 4.7. AGYW with *L*. *crispatus*-dominant microbiota (42.4%) generally had the lowest cytokine concentrations compared to women with more diverse microbiota (34/44 significantly upregulated cytokines). Frequencies of CCR5+CD4^+^ T-cells co-expressing CD38 and HLA-DR correlated positively with interleukin (IL)-6, TNF-α, GRO-α, macrophage inflammatory protein (MIP)-1α, and IL-9. While endogenous oestrogen had an immune-dampening effect on IL-6, TNF-related apoptosis-inducing ligand (TRAIL) and IL-16, injectable hormone contraceptives (DMPA and Net-EN) were associated with significantly lower endogenous hormone concentrations (p<0.0001 for oestrogen and progesterone) and upregulation of 34/44 cytokines. Since genital inflammation and the presence of activated CD4+ T cells in the genital tract have been implicated in increased HIV risk in South African women, the observed high levels of genital cellular activation and cytokines from AGYW may point towards biological factors increasing HIV risk in this region.

## Introduction

More than seventy percent of all new HIV infections occur in sub-Saharan Africa (SSA), with young women (aged 15–24) being three times more likely to become HIV-infected than their age-matched male counterparts [1,2]. Several socio-behavioural factors have been proposed to influence these high incidence rates of HIV in SSA, including gender inequality, limited access to sexual and reproductive health services, and age disparity between sexual partners [2]. However, these factors cannot fully explain the higher HIV prevalence seen in young women and it is likely that biological factors contribute to risk.

We have previously shown that the genital tract chemokines interferon gamma-inducible protein (IP)-10, macrophage inflammatory protein (MIP)-1α, MIP-1β, and interleukin (IL)-8 were associated with >3-fold higher HIV risk in women [3]. Furthermore, elevated genital cytokines were associated with significant alterations in genital epithelial barrier function and protease activity, as well as increased recruitment of activated CD4^+^ T-cells in African women [4]. Studies have since shown that the vaginal microbial dysbiosis are important drivers of genital inflammation, where women with a genital subtype dominated by *Gardnerella vaginalis* or *Prevotella* spp. [commonly associated with bacterial vaginosis (BV)] had the highest genital inflammatory profiles [5]. Compared to women from the United States, African women have higher frequencies of activated cervical T-cells [6] and African American women had higher genital pH than their white American counterparts (pH 4.7 vs 4.2) [7], more diverse vaginal microbiota, and lower relative abundance of *Lactobacillus* spp. (particularly *L*. *crispatus*) [7–9].

Adolescence is a developmental phase defined by significant hormonal changes that influence sexual maturity, and it is therefore likely that adolescent girls and young women (AGYW) differ anatomically, physiologically and, possibly, immunologically to adult women, which may impact on their risk of HIV infection. AGYW were more likely to have cervical ectopy and more variable vaginal microbiota than older women [10,11]. Studies have shown that AGYW with an immature epithelium (abundant columnar and metaplastic epithelium) tended to have higher genital concentrations of IL-1α, IL-1β, IL-6, IL-8, MIP-1α, RANTES (regulated upon activation, normal T-cell expressed and secreted), tumour necrosis factor (TNF)-α, IL-10, IL-12, and interferon (IFN)-γ than those with a mature epithelium [12] and that younger age was independently associated with increased cervicovaginal immune cell numbers [13].

AGYW in SSA represent a key HIV risk group, with unique factors that may influence their risk [2,14,15]. We hypothesised that African AGYW do not perfectly fit the internationally accepted definitions of vaginal health (defined by Amsel and Nugent criteria; [16,17]) and healthy AGYW from unique locations even with the same country in Africa may exhibit distinct variation in biological factors that could influence their HIV risk, using genital inflammation [18], HIV target cell activation [6], and vaginal *Lactobacillus*-dominance [5,19,20] as biomarkers. To test this hypothesis, we conducted an observational cohort study in healthy 16-22-year-old AGYW from Cape Town and Soweto in South Africa with the aim of characterising the healthy reproductive immune and microbiological environment in young women from SSA. Given that AGYW are likely to have only recently become sexually active, we evaluated the influence of hormone contraceptive (HC) choices and endogenous hormone levels on mucosal biomarkers of HIV risk.

## Methods and materials

### Ethics statement

The Human Research Ethics Committees of the Universities of Cape Town (UCT HREC #267/2013) and Witwatersrand (WITS HREC #M130745) approved this study. Women who were ≥18 years old provided written informed consent and those <18 years provided written assent, and consent was obtained from their parent(s) or legal guardian(s).

### Cohort description and screening for STIs/BV

Two hundred and ninety-eight HIV-negative, healthy, sexually active adolescents between the ages of 16 and 22 years from Cape Town and Soweto were recruited for this study, as previously described [21]. Women from Cape Town were recruited the Desmond Tutu HIV Foundation, which offers family planning services while women from Soweto were recruited from community outreach programs through the Perinatal HIV Research Unit. AGYW were excluded if they were HIV-positive, if they were pregnant, if they were menstruating at time of sampling, if they had been sexually active, had douched or used spermicides in the last 2 days, or if they had been on antibiotics in the last two weeks. All participants had passed menarche. Women were scheduled for their visits two weeks after their injection if they were on injectable contraceptives, or during the luteal phase of their menstrual cycle (day 14–28; self-reported). Women in the Cape Town arm were followed longitudinally for up to three visits. Women using depot medroxyprogesterone acetate were seen for their two follow-up visits every three months, while women using norethisterone enanthate (Net-En), combined oral contraceptives (COC) or Nuvaring were recalled for their follow-up visits every two months. Women were tested for STIs (*Chlamydia trachomatis*, *Neisseria gonnorrhoea*, *Trichomonas vaginalis*, *Mycoplasma genitalium*, Herpes Simplex Virus (HSV)-1, HSV-2, *Haemophilus ducreyi*, *Treponema pallidum*) using real-time multiplex PCR [22], BV (Nugent score) and candidiasis (by microscopy) [21]. Women positive for any of the abovementioned STIs, BV or candidiasis were excluded in this sub-analysis. Genital pH was measured from a lateral wall swab fluid using pH strips (pH range: 3.6–8.2; Macherey-Nagel, Düren, Germany).

### Measurement of endogenous sex hormones

Concentrations of oestrogen, progesterone and luteinising hormone (LH) were measured from blood plasma using electrochemiluminescence immunoassays (Cobas). Plasma was obtained two weeks after their injection for women using Net-En or DMPA or during the luteal phase of the menstrual cycle for all other women.

### Measurement of cytokine concentrations by Luminex

Luminex flow cytometry was used to measure genital cytokine concentrations in cervicovaginal mucus collected by Softcup (stored at -80°C and thawed). All samples were filtered by centrifuging at 1950g for 10 minutes at 4°C in SPIN-X 0.2μm cellulose acetate filters to exclude debris and to avoid technical issues during acquisition such as bead aggregation. The concentrations of 44 cytokines were measured using two different types of kits: the Human Cytokine Group I 27-plex [measuring the concentrations of fibroblast growth factor (FGF)-basic, Eotaxin, granulocyte-colony stimulating factor (G-CSF), granulocyte-macrophage colony-stimulating factor (GM-CSF), IFN-γ, IL-1β, IL-1RA, IL-2, IL-4, IL-5, IL-6, IL-7, IL-8, IL-9, IL-10, IL-12(p70), IL-13, IL-15, IL-17, IP-10, monocyte chemoattractant protein (MCP)-1, MIP-1α, MIP-1β, platelet-derived growth factor (PDGF)-BB, RANTES, TNF-α, and vascular endothelial growth factor (VEGF)] and the Human Cytokine 21-plex kit [measuring IL-1α, IL-2RA, IL-3, IL-12(p40), IL-16, IL-18, chemokine cutaneous T-cell-attracting chemokine (CTACK), growth regulated oncogene (GRO)-α, hepatocyte growth factor (HGF), IFN-α2, leukemia inhibitory factor (LIF), MCP-3, macrophage colony-stimulating factor (M-CSF), macrophage migration inhibitory factor (MIF), monokine induced by IFN-γ (MIG), β-nerve growth factor (NGF), stem cell factor (SCF), stem cell growth factor (SCGF)-β, stromal cell-derived factor (SDF)-1α, TNF-β, and TNF-related apoptosis-inducing ligand (TRAIL)] (Bio-Rad Laboratories Inc). Experiments were conducted according to the manufacturer’s instructions. The lower detection limits for the cytokines measured ranged from 1–307 pg/ml. Cytokine concentrations were determined from 5PL regression lines. All values below the detection limit were recorded as half of the lowest measured concentration for each cytokine. Samples were randomly allocated on the different assay plates, with ten samples selected for QC and included in all plates: Inter- and intra-plate correlations (Spearman correlations) between these ten samples were carried out for each cytokine amongst all the plates to confirm that the data were comparable. IL-15 was excluded for all analyses as the values did not pass QC. The cytokines IL-2, IL-5 and RANTES were reported as binary variables (presence/absence) as 76.0%, 64.2% and 52.6% of the values respectively were below detectable range. For certain analyses (where indicated), individual cytokines were grouped according to their functional classes using factor analyses to decrease the number of variables in a model, and to avoid multicollinearity.

### Measuring T-cell activation by flow cytometry

An eight-colour polychromatic flow cytometry panel was used to measure the level of expression of the HIV co-receptor CCR5, the level of activation (CD38 and HLA-DR) and proliferation (Ki67) in CD4^+^ and CD8^+^ T-cells isolated from cervical cytobrush-derived mononuclear cells (CMCs). A dump channel was added to the antibody panel to exclude dead cells, B-cells and monocytes (using VIVID, CD14 and CD19, respectively). Fluorescence Minus One controls (FMOs) were generated using PBMCs and validated on CMCs. The gating strategy is included in S1 Fig. Flow cytometry analysis was only performed on fresh cytobrush samples from the Cape Town cohort since live CMC staining was not possible for the Soweto arm. Cells were stained for flow cytometry according to methods described previously [23,24]. Compensation tubes were prepared each day. Data was analysed using FlowJo (version 10.0.8; Treestar, Ashland, OR). Gating was based on the FMO controls. Samples with <100 CD3+ events were excluded from further analyses [25].

### Bacterial 16S rRNA gene sequencing of genital microbiota

Lateral vaginal wall swabs (stored at -80°C upon receipt at the laboratory on the day of sampling) were thawed and treated with mutanolysin (25kU/ml, Sigma Aldrich), lysozyme (450kU/ml, Sigma Aldrich), and lysostaphin (4kU, Sigma Aldrich), then mechanically disrupted with a bead-beater (Thermo Savant FastPrep 120 Cell Disrupter system for 3x30 seconds at speed setting 5.5 m/s). DNA was extracted using the Mobio Powersoil DNA Isolation kit (Mo Bio Laboratories Inc) and the V4 hypervariable region of the bacterial 16S rRNA genes was amplified by PCR and sequenced on the Illumina MiSeq platform (300 bp paired-end) with the v3 chemistry as described in Lennard et al.[19] Only samples with ≥ 5000 reads were used for downstream analyses. The operational taxonomic unit (OTU) table was standardized (i.e. transformed to relative abundance * median sample read depth) and filtered such that each OTU had at least 10 counts in at least 2% of samples, or a relative abundance of at least 0.0001%.

For certain analyses, AGYW were categorized into three community types (CT) based on their microbiota. Women with an *L*. *crispatus*-dominant microbiota were categorised as CT3, those with an *L*. *iners*-dominated microbiota were categorised as CT2 and women with a more diverse genital microbiota dominated by species other than *Lactobacillus* spp (<50% any *Lactobacillus* spp) were classified as CT1.

### Statistical analysis

Statistical analyses and linear regressions were carried out using STATA 12.0. Graphs and figures were generated using Prism 6. The Mann-Whitney U-test was used to compare groups of continuous variables and two-sided Fisher’s exact tests for categorical variables. For three or more groups, the Kruskal-Wallis one-way analysis of variance was used. Statistical analyses were adjusted for multiple comparisons using false discovery rate step down procedures as described in Columb and Sagadai [26]. Microbiota statistical analyses were carried out in R using the packages phyloseq [27], metagenomeSeq [28], and Non-negative Matrix Factorization (NMF) [29]. 95% confidence intervals and p values of ≤0.05 were used to assess statistical significance.

## Results

Two hundred and ninety-eight sexually-active black AGYW from two socioeconomically disadvantaged communities in Cape Town (Masiphumelele) and Johannesburg (Soweto), South Africa were enrolled [21]. In these areas with a high HIV, STI and BV burden, only 90 (30.2%) were negative for common STIs (*C*. *trachomatis*, *N*. *gonorrhoea*, *T*. *vaginalis*, *M*. *genitalium*, HSV-2), candidiasis, or BV (Nugent 7–10), and were included in this study on vaginal health (35 from Cape Town and 55 from Soweto) (Table 1). Nineteen of the AGYW had Nugent scores between 4–6 but were included in this analysis as they would be considered clinically healthy and not receive treatment according to South African syndromic management guidelines. The median time from sexual debut for this cohort of AGYW was two years (IQR 1–3), their median number of lifetime partners was two (IQR 1–3), and 30% had been pregnant at least once. Socio-behavioural characteristics of AGYW did not differ significantly between Cape Town and Johannesburg [21], and did not differ significantly from those with BV or an STI. Sexual behaviour in this cohort was also previously described by Barnabas et al. (2018). The 90 AGYW in this study tended to have similar number of lifetime partners, similar numbers of AGYW reported using soap or another product applied to their genital region as part of their hygiene practices, as well as products inserted into their vagina’s (including traditional medicines, drying agents, douching, cloths, paper) [21].

**Table 1:** Characteristics of adolescents negative for STIs, BV and candidiasis *.

Characteristics	All participants	Cape Town	Johannesburg	p value
N	90	35	55	
Age	18 [17–20]	18 [17–20]	18 [17–20]	0. 2312
BMI	22.48 [20.57–26.40]	23.14 [20.83–26.77]	22.15 [20.57–26.40]	0.7125
Endogenous hormones				
Oestrogen (pmol/L)	62.7 [95.8–320.5]	72.9 [51.8–95.2]	266 [89–506]	**<0.0001**
Progesterone (nmol/L)	1.25 [0.8–2.1]	0.9 [0.5–1.5]	1.25 [1.2–3.7]	**0.0006**
Luteinising hormone (IU/L)	4.65 [2.8–8.7]	4.4 [2.4–6.8]	6.1 [3.1–9.5]	0.1112
Hormone Contraceptive:				**<0.0001**
Net-En	37/89 (41.6%)	25/35 (71.4%)	12/54 (22.2%)	
DMPA	14/89 (15.7%)	7/35 (20.0%)	7/54 (13.0%)	
COC	4/89 (4.49%)	2/35 (5.71%)	2/54 (3.70%)	
Nuvaring	1/189 (1.12%)	1/35 (2.86%)	-	
Male condom only	28/89 (31.5%)	-	28/54 (51.9%)	
None	5/89 (5.62%)	-	5/54 (9.26%)	
Vaginal pH	4.7 [4.4–5.3]	4.4 [4.1–5]	4.7 [4.4–5.3]	**0.0417**
Ever been pregnant	20/67 (29.8%)	7/30 (23.3%)	13/37 (35.1%)	0.421

* Values were either reported as medians [and interquartile ranges] or as proportions (and precentages).

### Endogenous and exogenous hormone levels in AGYW

The majority of AGYW were using the injectable progestin-only HCs, with 37/90 using Net-En and 14/90 using DMPA (Table 1). There were differences in contraceptive choices between Cape Town and Johannesburg [21], and younger adolescents (16–17 years) were more likely to use condoms only while older AGYW (18–22 years) were more likely to use HCs (Fisher’s exact p = 0.006).

To account for the influence of the menstrual cycle on hormone levels at the mucosa, we scheduled study visits during the luteal phase for women not on injectable HCs. In adolescents not using HCs (33/90), plasma progesterone concentrations were significantly lower in adolescents than the reported reference ranges for adults (1.25nmol/L versus 36 nmol/L; p<0.001), while oestrogen concentrations (95.75 pmol/L [62.7–320.5]) also tended to be lower (161–774 pmol/L), although not significantly (p = 0.320).

Endogenous hormone concentrations were significantly lower in AGYW using injectable progestin-only HCs compared to those not using HCs: progesterone concentrations were 3.6- and 3.2-fold lower in DMPA or Net-EN users than non-HC users, respectively (p<0.0001 for both); LH levels were 1.9- to 2-fold lower in DMPA and Net-EN users (p = 0.0037 and p = 0.017 respectively); while oestrogen was 11.2- and 5.1-fold lower in DMPA and Net-EN users, respectively (p<0.0001 for both; S2 Fig).

### Genital cytokine profiles in healthy adolescents

**Fig 1:**
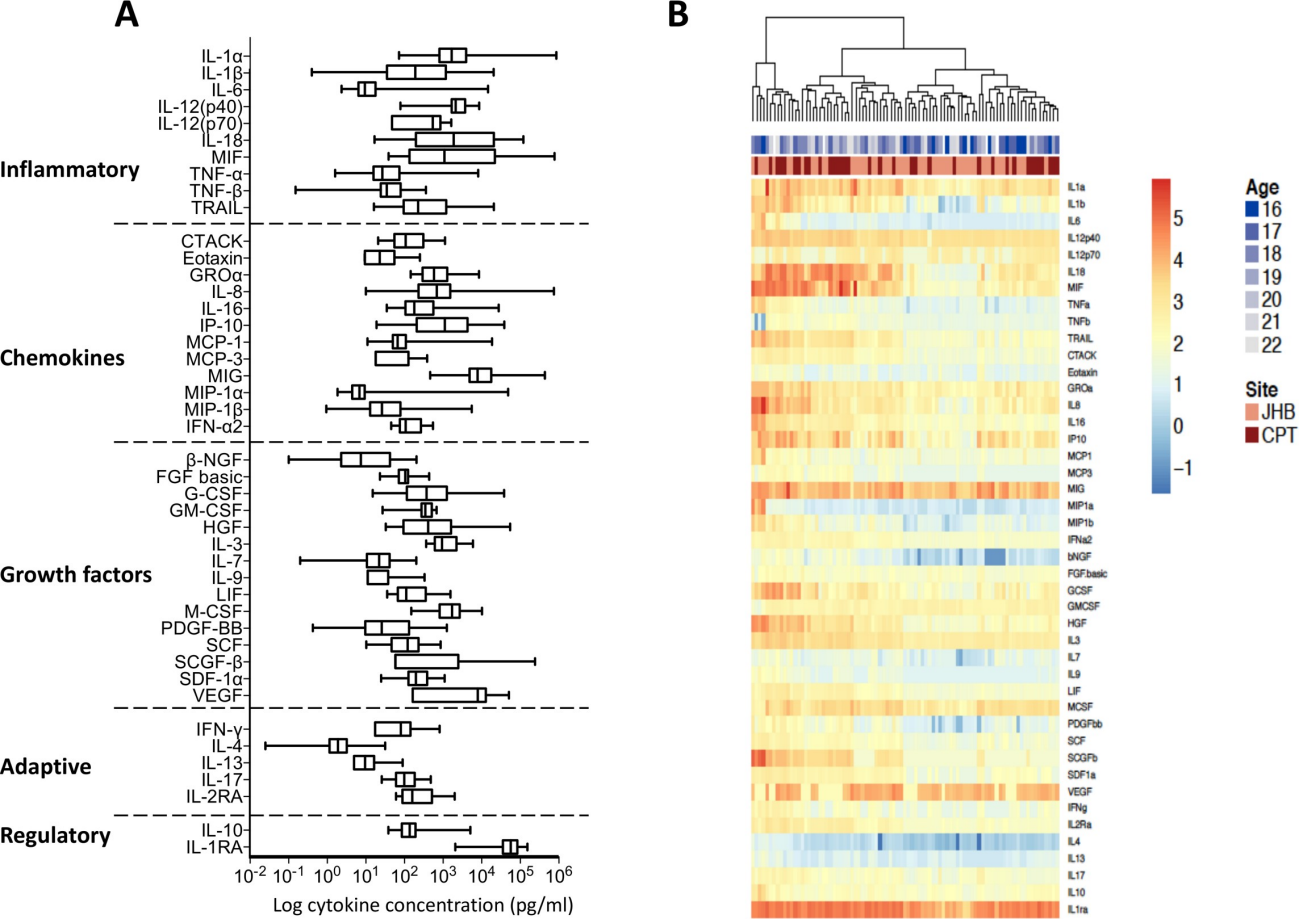
Concentrations of cytokines in genital secretions from reproductively healthy AGYW from South Africa. **A.** Box-and-whisker plots showing the median cytokine concentration and 95% confidence intervals for all cytokines measured, grouped by functional classes (inflammatory cytokines, chemokines, growth factors, adaptive and regulatory cytokines respectively). **B.** Unsupervised hierarchical clustering of all measured genital cytokines in healthy women. The site is represented by light (Cape Town) and dark red (Johannesburg), while ages are represented by the shades of blue.

Genital cytokine concentrations were measured in cervicovaginal secretions collected during the luteal phase of the menstrual cycle (in those not using HCs or using combined oral contraceptives) or two weeks after their last injection in those using long-acting injectable HCs (Fig 1), to account for possible menstrual cycle fluctuations in cytokine concentrations. We hypothesized that AGYW from Cape Town and Johannesburg would be exposed to similar age-associated risk factors for HIV and therefore have largely comparable genital cytokine profiles. In support of this, no distinct separation in cytokine profiles between AGYW cohorts from Cape Town and Johannesburg were found by unsupervised hierarchical clustering (Fig 1B).

In an unadjusted comparison, oestrogen was associated with the downregulation of three cytokines—the inflammatory cytokines TRAIL (p = 0.04), IL-6 (p = 0.024) and the chemokine IL-16 (p = 0.024)—after correcting for the participant age, hormonal contraceptive use and semen exposure (S3 Fig). Statistical significance was however lost after adjusting for multiple comparisons. Progesterone and LH did not follow a similar trend.

**Fig 2:**
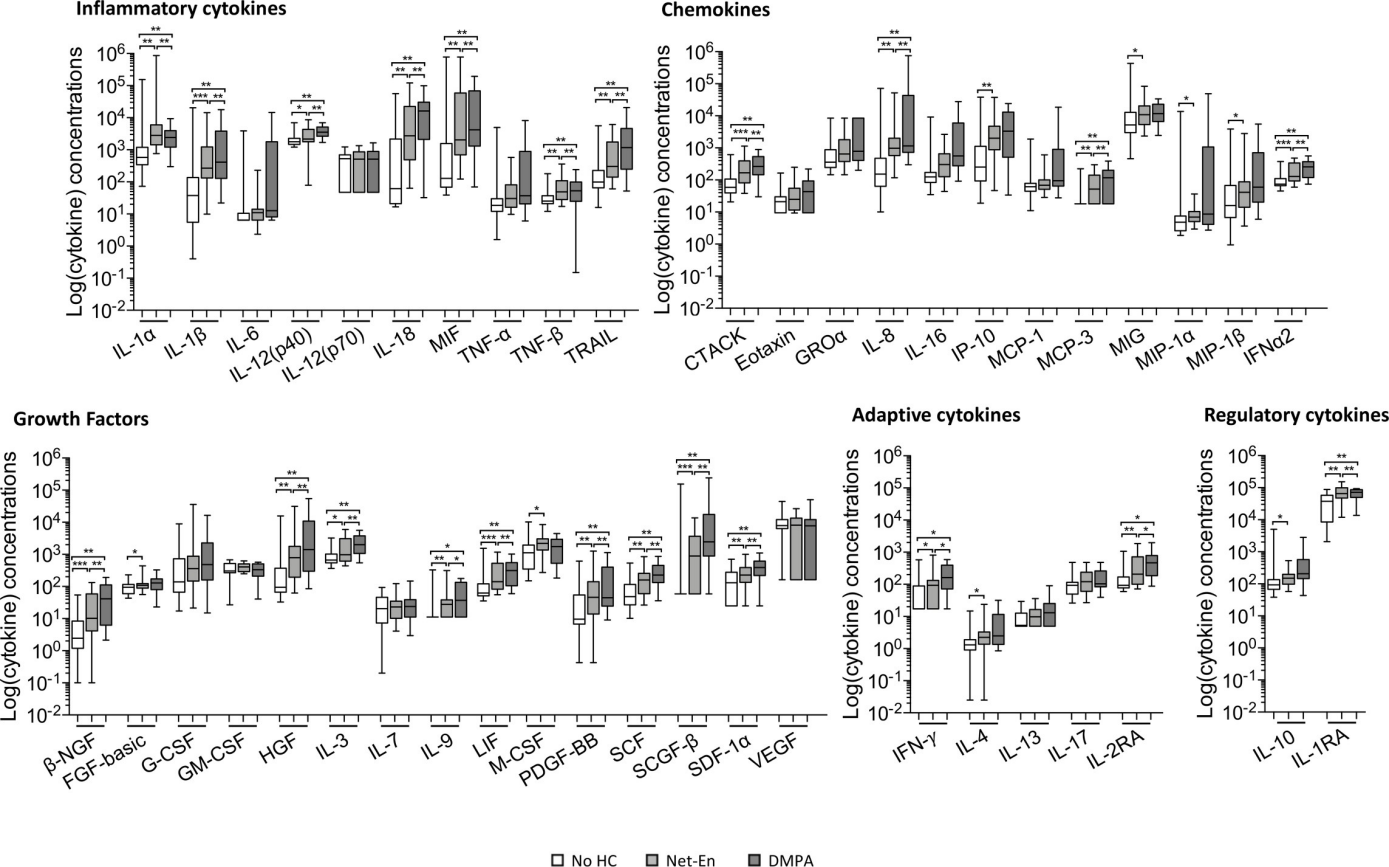
Comparison of cytokine concentrations in adolescents not using HC (white bars), those using Net-En (light grey bars) and those using DMPA (dark grey bars). Mann Whitney U-tests were used to compare groups and a p ≤0.05 was considered significant, after adjusting for multiple comparisons. *p<0.05; **p<0.01 and ***p<0.001. The box-and-whisker plots show the median, IQR and 5–95% range.

On the other hand, injectable HC use had a significant effect on genital cytokines across several functional classes (Fig 2). After adjusting for multiple comparisons, adolescents using Net-En had significantly increased concentrations of more than 70% of cytokines measured (including inflammatory cytokines: IL-1α, IL-1β, IL-12(p40), IL-18, MIF, TNF-β, TRAIL, chemokines: CTACK, IL-8, IP-10, MCP-3, MIG, MIP-1α, MIP-1β, IFN-α2, growth factors: β-NGF, FGF-basic, HGF, IL-3, IL-9, LIF, M-CSF, PDGF-BB, SCF, SCGF-β, SDF-1α, adaptive cytokines: IFN-γ, IL-4, IL-2RA and regulatory cytokines: IL-10 and IL-1RA) compared to women not using HCs. Those using DMPA had significantly higher concentrations of ~50% of the cytokines, (including inflammatory cytokines: IL-1α, IL-1β, IL-12(p40), IL-18, MIF, TNF-β, TRAIL, chemokines: CTACK, IL-8, MCP-3, IFN-α2, growth factors: β-NGF, HGF, IL-3, IL-9, LIF, PDGF-BB, SCF, SCGF-β, SDF-1α, adaptive cytokines: IFN-γ, IL-2RA and regulatory cytokine: IL-1RA). Although more cytokines were statistically significantly elevated in Net-EN than DMPA users, cytokines upregulated by both types of HC were consistently higher in DMPA than Net-En users.

### Vaginal pH and microbiome in healthy adolescents

The overall median vaginal pH for healthy South African AGYW was 4.7 (IQR 4.4–5.3), with 89.9% having a vaginal pH >4.5. Adolescents with a Nugent score of 0–3 had a median vaginal pH of 4.7 (IQR 4.1–5) while those with intermediate microbiota (Nugent 4–6) had a median pH of 5.6 (IQR 4.7–5.6; p<0.001). Vaginal pH tended to vary significantly by site, with adolescents in Johannesburg having a higher median pH [4.7 (IQR 4.4–5.3)] compared to those in Cape Town [4.4 (IQR 4.1–4.5); p = 0.0415]. The number of adolescents with intermediate Nugent scores 4–6 did not differ significantly by site: 17.1% in Cape Town versus 23.6% in Johannesburg; p = 0.598.

**Fig 3:**
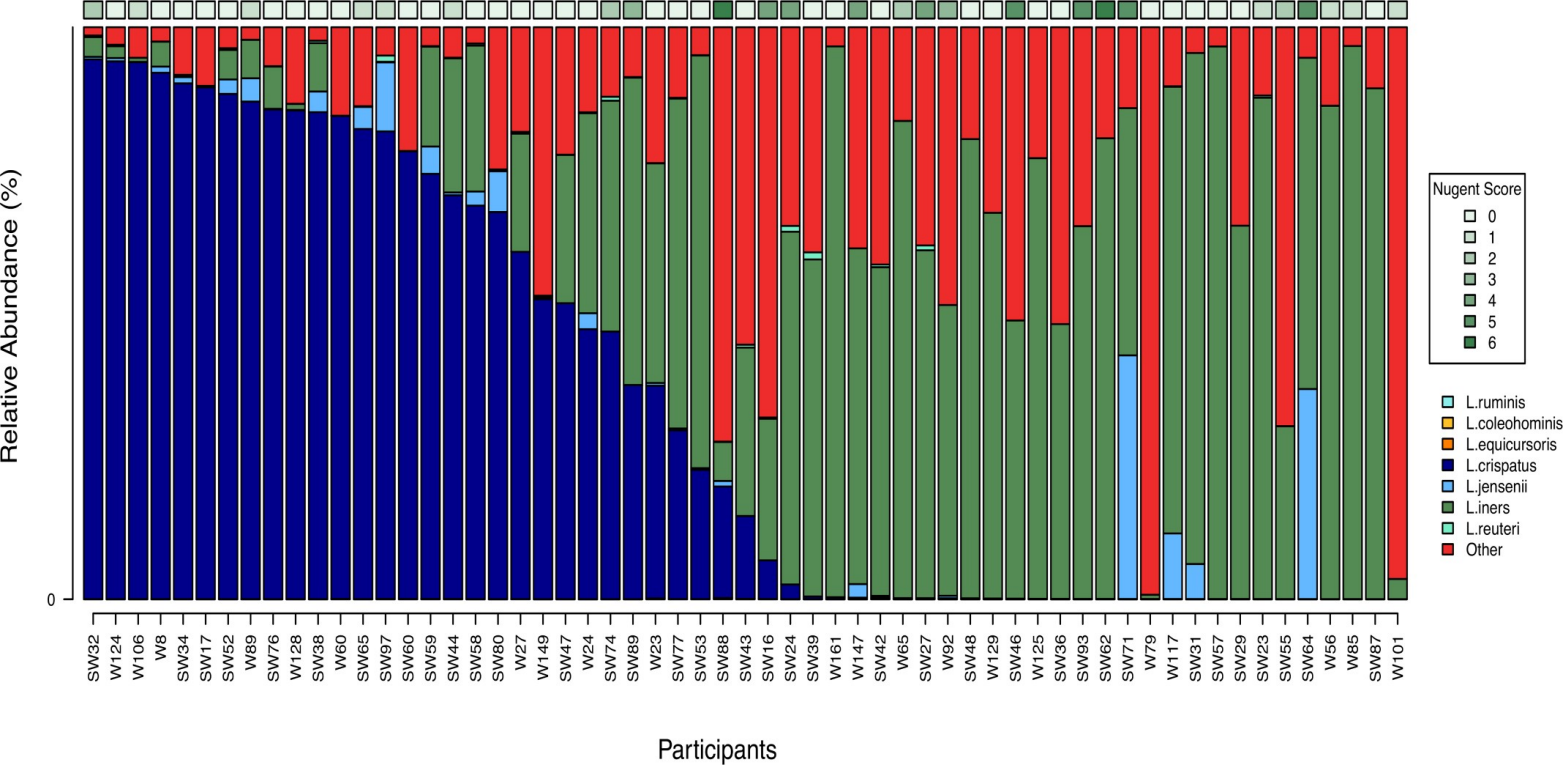
Relative abundance of the different vaginal bacterial species in each healthy adolescent. *L*. *crispatus* is shown in dark blue, *L*. *iners* in dark green, *L*. *ruminis*, *L*. *jensenii* and *L*. *reuteri* in shades of light blue, *L*. *equicursoris/delbrueckii* in dark orange and *L*. *coleohominis* is shown in light orange, while non-*Lactobacillus* spp. are shown in red for each participant. The Nugent score is shown above the figure in green.

**Fig 4:**
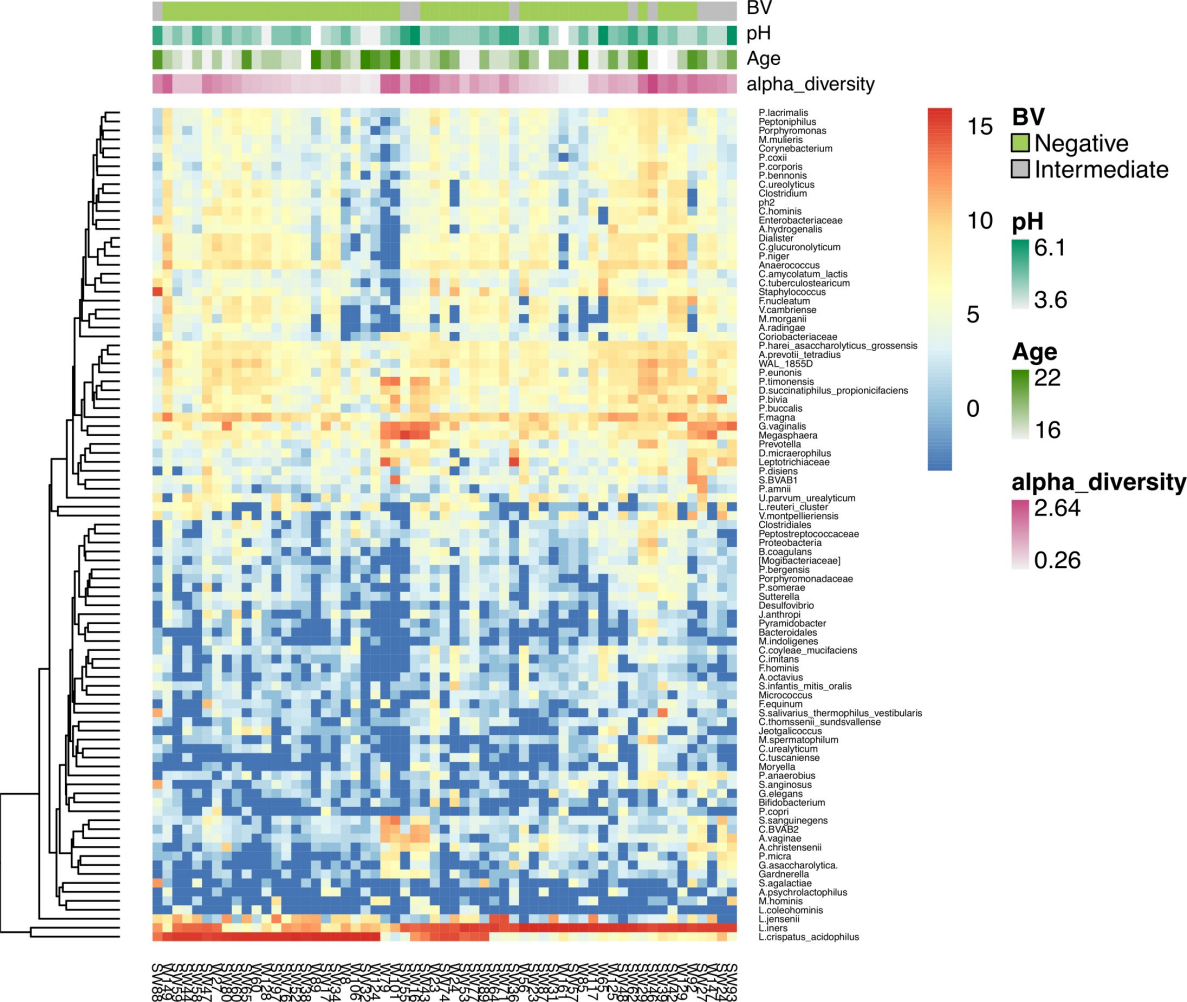
Unsupervised hierarchical clustering of bacteria merged at lowest taxa in the genital tract of young women. The top of the figure shows clustering according to BV status (light green: negative; grey: intermediate), vaginal pH (blue green), age (darker green) and alpha diversity (pink).

Of the 90 AGYW with no STIs or BV, 59 (65.5%) provided lateral vaginal wall swabs yielding sufficient DNA for 16S rRNA sequencing of the vaginal microbiota, of which 49 had Nugent scores 0–3 and 10 had Nugent scores 4–6 (Fig 3). More than 80% of healthy AGYW had vaginal microbiota dominated by *Lactobacillus* spp. (81.3%; 48/59), with 25/48 (52.1%) being dominated by *Lactobacillus* spp. other than *L*. *iners* (mostly *L*. *crispatus*; classified as Community type [CT] 3) [19] followed by L. iners (CT2). *L*. *crispatus* was found only in AGYW with Nugent scores 0–3 while *L*. *iners* was detected in both those with Nugent scores 0–3 and 4–6. AGYW with higher relative abundances of *L*. *crispatus* (CT3) tended to have lower alpha diversity compared to those with a higher relative abundance of *L*. *iners* (CT2), although not significantly (p = 0.06). Despite selecting for AGYW with no STIs or BV, 11/59 adolescents had a vaginal microbiota with low relative abundance of *Lactobacillus* spp., high alpha diversity, and high relative abundances of *G*. *vaginalis* and other BV-associated taxa including *Prevotella spp*, BVAB1, *Megasphera*, and *Sneathia* (Fig 4; CT1) [19]. Neither vaginal pH (p = 0.20), HC choice (ANOVA p = 0.59) nor age (p = 0.69), were associated with vaginal bacterial alpha diversity in AGYW with Nugent scores 0–3.

### Vaginal microbiota influence genital cytokines

**Fig 5:**
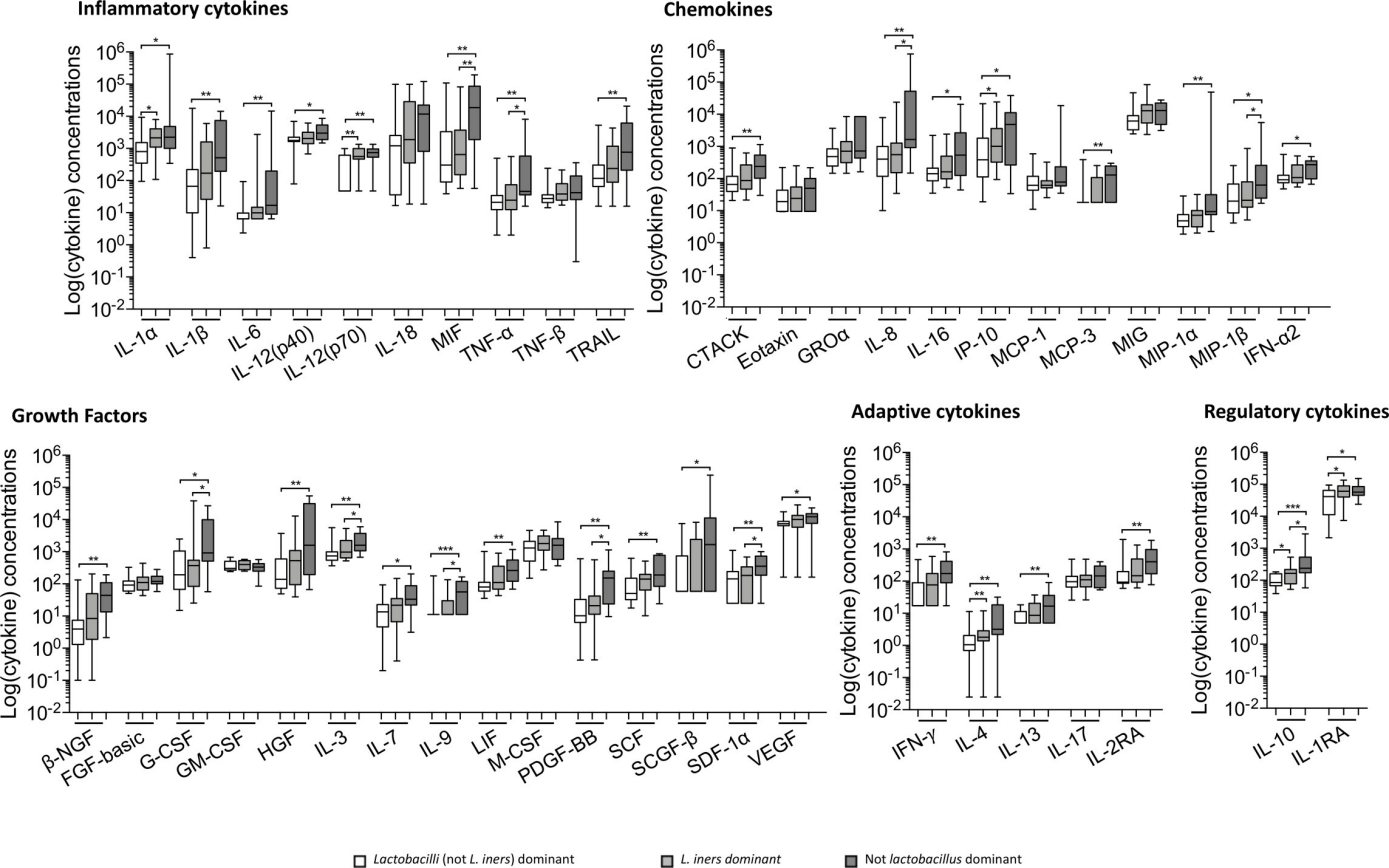
Genital cytokine concentrations among women with a microbiota dominated by non-*L*.*iners* lactobacilli (CT3; shown as white bars), women dominated by *L*. *iners* (CT2; shown with the light grey bars) and women having <50% lactobacilli in their microbiome (CT1; represented by the dark grey bars). Only differences remaining after correcting for multiple comparisons were shown in this figure. Mann Whitney U-tests were used to compare groups and a p value of ≤0.05 was considered significant.

AGYW with *L*. *crispatus*-dominant microbiota (CT3) tended to have lower genital cytokine concentrations compared to the women who were not *Lactobacillus* spp. dominant (CT1; Fig 5). Similarly, those with an *L*. *iners*-dominated microbiota (CT2) also tended to have a lower genital inflammatory profile than those with a low relative abundance of *Lactobacillus* spp. (CT1), although IL-1α, IL-12(p70), IP-10, IL-4, IL-10 and IL-1RA tended to be upregulated compared to the CT3 group, but not statistically significantly so. Women with more diverse CT1 microbiota (higher relative abundance of *G*. *vaginalis* and other BV-associated taxa) had elevated concentrations of 34/44 cytokines (including IL-1α, IL-1β, IL-6, IL-12(p40), IL-12(p70), MIF, TNF-α, TRAIL, CTACK, IL-8, IL-16, IP-10, MCP-3, MIP-1α, MIP-1β, IFN-α2, β-NGF, G-CSF, HGF, IL-3, IL-7, IL-9, LIF, PDGF-BB, SCF, SCGF-β, SDF-1α, VEGF, IFN-ɣ, IL-4, IL-13, IL-2RA, IL-10 and IL-1A) compared to those with *L*. *crispatus*-dominated CT3 microbiomes, although this was not significant after correcting for multiple comparisons. HC type was uniformly distributed across the three CT groupings and did not appear to influence cytokine levels (Fisher’s exact = 0.73).

### Genital T-cell activation in healthy adolescents

**Fig 6:**
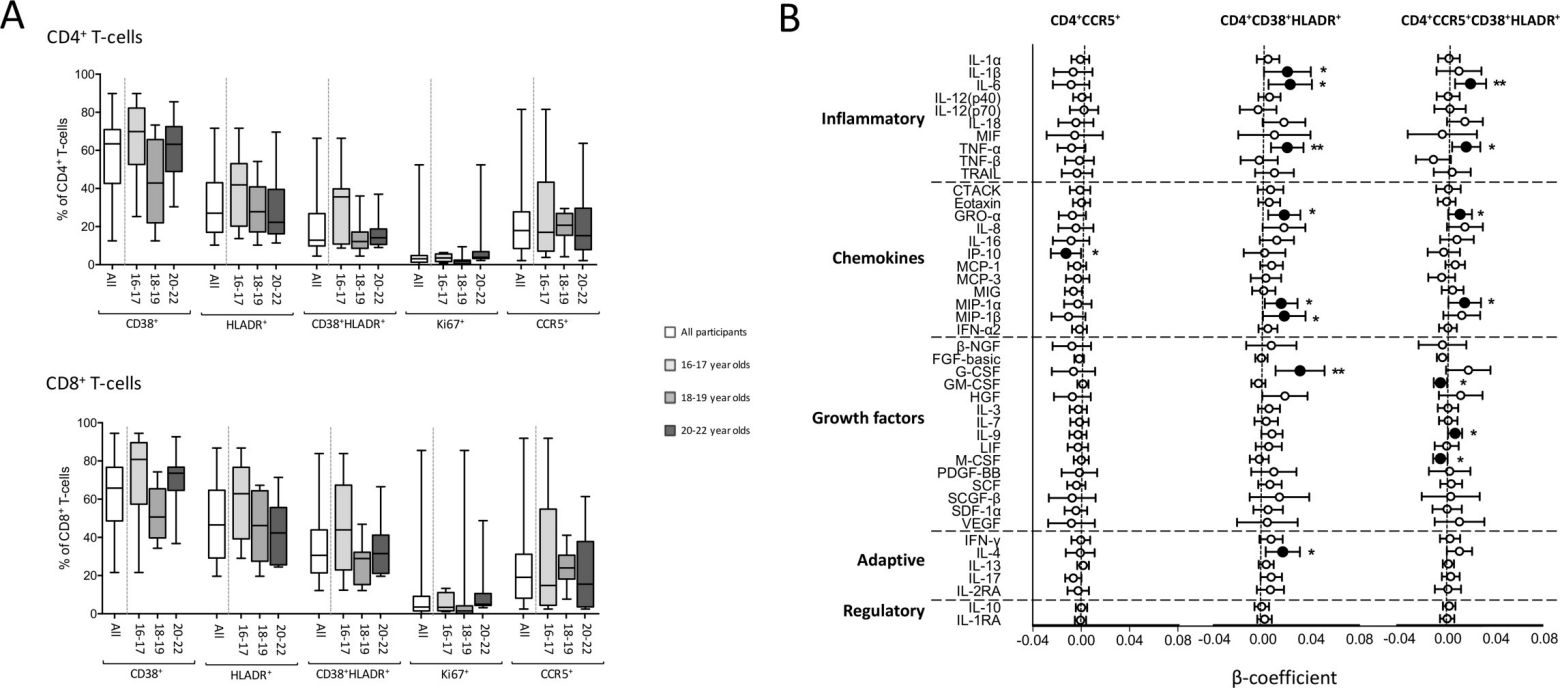
Comparison of CD4+ T-cell activation markers by age categories in in AGYW. **A.** The overall expression level for all participants are represented by the white bars, and the grey bars represent the expression levels split according to age. Mann Whitney U-tests were used to compare groups and a p value of ≤0.05 was considered significant. **B.** Linear associations between the different cytokines and frequencies of activated CD4+ T-cells. Each association is shown as a β-coefficient and the error bars are the 95% confidence interval. Statistically significant associations are shown in black and p value of ≤0.05 was considered significant.

Expression of the cellular markers CD38, HLADR, Ki67, and CCR5 by cervical cytobrush-derived CD4^+^ T-cells was measured from AGYW residing in Cape Town. S1 Fig shows the gating strategy used to determine activation levels by cervical T-cells. CCR5^+^ T-cells are thought to represent the preferred target cells for HIV infection [30] and we hypothesized that the level of expression of this marker may reflect susceptibility to HIV infection. Most of the CCR5^+^CD4^+^ T-cells present in the genital tracts of AGYW co-expressed CD38 or HLADR (72.8% and 39.5% respectively) (Fig 6). Approximately 25% of genital CCR5^+^CD4^+^ T-cells were highly activated (CD38^+^HLADR^+^) and could be potential targets for HIV infection. No significant difference in cellular marker expression in either cervical CD4^+^ or CD8^+^ T-cells was noted across age groups (Fig 6A).

### Genital cellular activation and genital cytokine profiles

We have shown that women with elevated levels of inflammatory cytokines and chemokines (including MIP-1α, MIP-1β, IP-10, IL-8, IP-10, MIP-1β, IL-8 and MCP-1) were at increased risk of HIV [18,31]. We hypothesized that chemotaxis of highly activated HIV target cells to the genital mucosa provide the underlying mechanism by which elevated genital cytokines increase HIV risk. To assess the relationship between cytokine markers of HIV risk and cellular targets of infection, cytokine concentrations in genital secretions and cervical cytobrush-derived T-cell activation in matching samples were compared using multivariate linear regression models (Fig 6B). After correcting for multiple comparisons, frequencies of highly activated CD38^+^HLA-DR^+^ cervical CD4^+^ T-cells were positively associated with MIP-1α (p = 0.03), MIP-1β (p = 0.049), growth regulated oncogene (GRO)-α (p = 0.011), IL-1β (p = 0.041), IL-6 (p = 0.015), TNF-α (p = 0.005), G-CSF (p = 0.004) and IL-4 (p = 0.024). Furthermore, IL-6 (p = 0.006), TNF-α (p = 0.012), GRO-α (p = 0.041), MIP-1α (p = 0.042), and IL-9 (p = 0.039) were significantly positively associated with frequencies of CCR5 on CD4^+^CD38^+^HLADR T-cells. While several classes of inflammatory cytokines and chemokines were positively associated with CD4+ T-cell frequencies, GM-CSF and M-CSF were negatively associated with CD4 T-cell activation (p = 0.036 and 0.05 respectively).

### Genital cellular activation, vaginal microbiota, and hormones

Matching cervical cellular activation and vaginal microbiota data were only available for a subset of AGYW from Cape Town (n = 21). No differences in frequencies of activated or proliferating CD4^+^ T-cells were evident by microbiota composition (comparing CT1, CT2 and CT3 microbiomes; S4 Fig).

Despite increased concentrations of certain cytokines in adolescents using Net-En and DMPA, the frequencies of cellular activation markers (CD38 and HLA-DR), CCR5, and proliferation marker Ki67 on CD4+ T-cells in AGYW using long-acting injectable HCs were similar to women using other HCs (S5 Fig). This was likely influenced by the small number of cytobrush samples available for this sub-analysis (Net-En: n = 6; DMPA: n = 28; Nuvaring/COC: n = 3) and therefore needs to be confirmed in future studies.

### Longitudinal characteristics of vaginal health in AGYW

**Fig 7:**
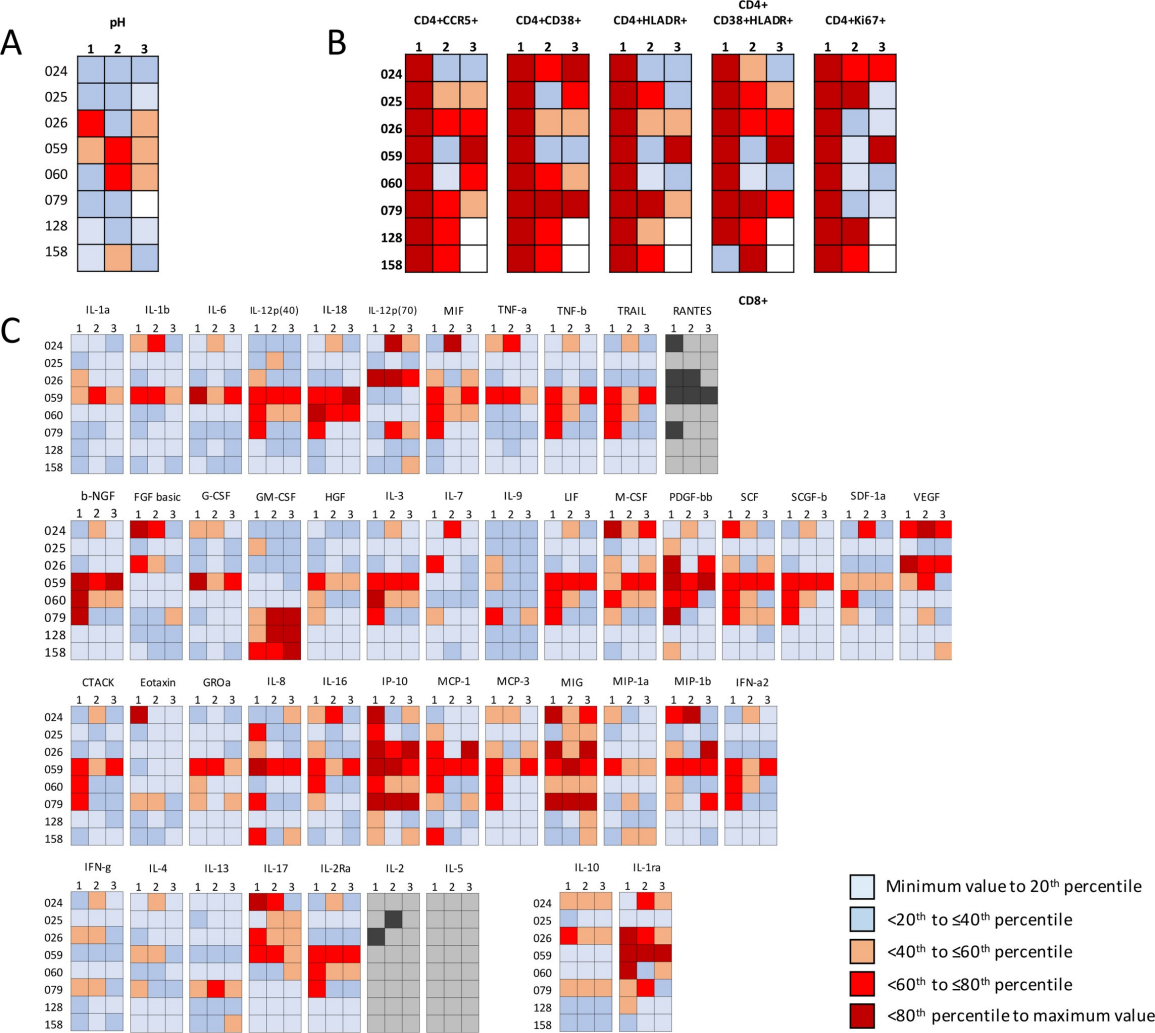
Longitudinal variation in genital pH, cytokine levels, and cervical cellular activation for AGYW who were persistently reproductively healthy over the three study visits. The percentile ranges were generated using the baseline values for the whole WISH cohort (n = 298). Blank squares represent missing data. RANTES, IL-2 and IL-5 are shown in grey as their concentrations were binarised as present (dark grey) or absent (light grey).

Of the 90 adolescents who were considered reproductively healthy at enrolment and included in this study, the 35 women from Cape Town were followed longitudinally for three visits over 4–6 months. 8/35 (28.9%) remained negative for BV, STIs and yeast infections over the three visits. In these persistently healthy AGYW, vaginal pH ranged from 3.6 to 5.3 over time (Fig 7A). Fig 7 shows that the genital tract milieu was quite variable even in the absence of any infection or dysbiosis (Fig 7B). Average variation within each participant over time was generally lower than the variation between healthy women at any point in time (coefficients of variation; S1 Table).

## Discussion

African AGYW are a key HIV risk group, although the reason for this vulnerability is poorly understood. We hypothesized that physiological factors associated with adolescence, menarche, and sexual debut are like to influence HIV risk in this group. To evaluate the healthy genital environment in AGYW from Africa in the context of factors that can influence HIV risk, we focused on vaginal pH, genital cytokine concentrations, cervical cellular activation and vaginal microbiota as biomarkers for risk. There was a very high burden of STIs and BV in these two at-risk cohorts of AGYW from two socio-economically deprived communities in Southern Africa, and only about 30% were negative for BV by Nugent criteria, inflammatory STIs and candidiasis. In this sub-group of healthy women, we found that injectable HC use and microbial dysbiosis increased genital inflammation, while endogenous oestrogen concentrations had an immune-dampening effect.

Adolescence is a period of significant hormonal change and rapid growth associated with a gain in reproductive maturity [32], often involving sexual debut and initiation of HC use [33]. Long-acting, injectable progestins are commonly used due to difficulty with adherence to daily medication at this age and because they are discreet. Several of these factors have been shown to increase the risk of HIV acquisition [33–35]. An increased risk of STIs has been reported around early-menarche [36,37], which suggests that characterization of sex hormone changes in these young women is important. In this study, AGYW not using HCs had significantly lower LH and higher progesterone concentrations than published adult reference ranges. Endogenous oestrogen, progesterone, and LH were significantly lower in adolescents using injectable HCs than those who did not. Synthetic progestins, like DMPA and Net-En, bind to the progesterone receptor and competitively inhibit natural progesterone binding [38]. DMPA and Net-En also prevent ovulation by suppressing LH [39]. The hypoestrogenic state induced by synthetic progestins has been described in adult women, where DMPA resulted in oestrogen levels similar to postmenopausal levels [40]. In these adolescents, a similar effect on endogenous oestrogen was also observed in adolescents using Net-En, another progestin-only injectable that is thought to have less detrimental effects than DMPA. Net-En was not shown to similarly lower oestrogen levels in studies conducted in South Africa [41,42]. Lower endogenous oestrogen concentrations in adolescents could impact genital health since decreased oestrogen has been associated with higher pH, decreased superficial epithelial maturity and imbalances in the vaginal microbiota, including a reduction in lactobacilli [42].

We found that endogenous oestrogen levels in AGYW had a significant immunomodulatory effect on IL-6, TRAIL and IL-16, which were lowest in adolescents with the highest oestrogen levels. While IL-6 and TRAIL are important inflammatory cytokines that regulate neutrophil recruitment and programmed-cell death respectively, IL-16 is a chemokine that recruits and activates T-cells to the mucosa that can enhance HIV infection through the NFkB pathway [43,44]. The effect of endogenous oestrogen was strongest in the older (20-22-year-old) adolescents, where it was associated with significant downregulation of cytokines across all functional classes: inflammatory cytokines (IL-12(p40), MIF, TNF-β, TRAIL), chemokines (CTACK, IP-10, IFN-α2), growth factors (IL-3, LIF, PDGF-BB, SCF), adaptive and regulatory cytokines (IL-2RA and IL-1RA, respectively). This concentration-dependent effect of oestrogen has previously been reported by Straub et al. (2007) in adult women, where higher levels of oestrogen had anti-inflammatory effects on cell-mediated immune responses but could induce inflammatory cytokines such as TNF-α, IL-6 and IL-1β at lower concentrations [45].

In contrast to the immunomodulatory properties noted with endogenous oestrogen, we found that injectable HC use was associated with markedly upregulated cytokines of all functional classes, especially inflammatory cytokines and chemokines, which could influence HIV/STI risk. A recent study in young South Africans showed that elevated progestin levels in women using DMPA or Net-En was associated with higher HIV target cell frequencies and a significantly higher risk of HIV acquisition.[46]

Most of the healthy African adolescents included in this study had vaginal microbiota dominated by *Lactobacillus* spp, similar to American and European adult women, with the most dominant vaginal species being *L*. *iners* (CT2) and *L*. *crispatus* (CT3) [47,48]. Adolescents with lactobacilli-dominated vaginal CT3 microbiota (non-*L*. *iners*) had the lowest genital cytokine levels while those with vaginal microbiota not dominated by lactobacilli (CT1) had the highest genital cytokine concentrations. This suggests that, even in women considered to have no STIs, BV or candidiasis, microbes causing vaginal dysbiosis may lead to increased inflammation and HIV risk [5,19,20,49]. Lactobacilli have the capacity to dampen immune responses triggered by cervicovaginal epithelial cells which could be, in part, due to lactic acid production [50]. Genital lactic acid, predominantly produced by lactobacilli species, was shown to induce an anti-inflammatory response from cervicovaginal epithelial cells through the production of IL-1RA, and the inhibition of IL-6, IL-8, TNF-α, RANTES, and MIP-3α [51]. Hydrogen peroxide-producing lactobacilli have also been associated with lower levels of the inflammatory cytokine IL-1β [52]. Different lactobacilli species appear to be associated with different inflammatory outcomes: women with *L*. *crispatus* had lower genital cytokine levels [49,53], and decreased risk of HIV acquisition[54] and transmission [9] compared to women with a higher relative abundance of *L*. *iners*.

Activated CD4+CCR5+ T-cells are thought to be the preferred targets for HIV in the genital tract [30]. More than 70% of CD4+CCR5+ T-cells in the genital tract of AGYW co-expressed the activation marker CD38, and about a quarter were highly activated (co-expression of both CD38 and HLADR). A high proportion of these cells could, in part, explain the higher susceptibility of these young women to HIV. In this cohort, younger women (16-17-years) tended to have higher cellular activation levels than their older counterparts (20–22 years), although this was not significant. Achilles et al. (2016) have shown that 18-21-year-old American women had higher activated genital CD4+ T-cell numbers compared to the older women (22-27-year olds) [55].

An important finding from this study was that activated cervical T-cells were broadly positively associated with several functional classes of genital cytokines, particularly for HIV target cells like CD4^+^CD38^+^ and CD4^+^CD38^+^HLADR^+^ T-cells, and CD4^+^CD38+HLADR^+^ T-cells expressing CCR5^+^. Mucosal inflammation alters HIV susceptibility and could increase risk through recruitment of activated immune cells and upregulation of viral receptors on immune cells in the genital mucosa [56]. Not only are activated CD4^+^CCR5^+^ T-cells preferential HIV targets but they also induce cytokines and can recruit additional target cells [57,58].

In a subset of AGYW who remained BV, STI and candida negative during follow-up, vaginal pH, inflammation, and cellular activation varied within each participant as well as between participants. Interestingly, those who remained STI/BV/candidiasis-negative had genital cellular activation levels that were higher than the cohort average, while maintaining relatively low genital inflammation levels. This would suggest that being healthy in the South African context cannot solely be defined by low cellular activation levels in the genital tract.

One of the limitations of the study was the fact that cellular activation levels /target cell frequencies were only available in the Cape Town arm of the cohort and not the Soweto arm, due to logistical reasons. Also, women with an HPV infection were not excluded from this study. However, previous data from our lab showed that, in sub-Saharan African women, HPV infections did not influence genital inflammation levels [59] and would not be a potential confounder in the analyses presented in this paper. HSV-2 serology was only measured in the Cape Town arm of the cohort and HSV-2-seropositive women were not excluded. While HSV-2 serology has been previously associated with increased genital cytokine levels, only 3/35 women were HSV-2 seropositive, and unlikely to skew the observed associations between the different factors investigated and genital inflammation levels.

In conclusion, healthy adolescents from two centres in South Africa were shown to have different endogenous hormone profiles compared to adults, which was skewed further in those using HCs. Although these healthy adolescents had genital microbiota dominated by lactobacilli, particularly *L*. *crispatus* (CT3) and *L*.*iners* (CT2), ~90% had a vaginal pH >4.5, considered to be an indication of BV. Genital cytokine concentrations, which were positively associated with frequencies of cervical CD4+ T-cell activation and genital microbial dysbiosis (CT1), were influenced by endogenous and exogenous hormones, particularly the long-acting injectable contraceptives Net-EN and DMPA. Together, the data provide insight into important biological factors that may contribute to higher HIV risk in adolescent South African women, even when considered reproductively healthy.

## Supporting information

S1 Fig Flow cytometry gating strategy used to measure the expression of the cellular markers CCR5, Ki67, CD38, and HLA-DR on CD4+ and CD8+ T-cells. The position of gates for all markers were based on FMOs.
(TIFF)

S2 Fig Endogenous hormone levels (progesterone, oestrogen and luteinising hormone) in AGYW not using HC, women using Net-En, and those using DMPA. The median concentration of hormone for each group is shown by the solid line. A p value of ≤0.05 was considered significant.
(TIF)

S3 Fig Multivariate linear regressions showing the associations between oestrogen, progesterone and luteinising hormone, and cytokines. Participant age, hormonal contraceptive use and semen exposure were corrected for. Each association is shown as a β-coefficient and the error bars are the 95% CI. Statistically significant associations are shown in red and p value of ≤0.05 was considered significant.
(TIF)

S4 Fig Frequencies of activated/proliferating CD4^+^ T-cells by microbiota composition. CT1 is shown by red dots, and CT2 and CT3 by light blue and dark blue dots. A p value of ≤0.05 was considered significant.
(TIF)

S5 Fig Frequencies of cellular activation markers (CD38 and HLA-DR), CCR5, and proliferation marker Ki67 on CD4+ T-cells in women using injectable HCs and other HCs. Women using Net-En are shown in blue, women using DMPA in red and those using COC or Nuvaring are shown in green. A p value of ≤0.05 was considered significant.
(TIF)

S1 Table Coefficients of variation (%) between healthy women at visit 1, 2 and 3 and among women who remained healthy over the three visits. (DOCX)

S2 Table Raw cytokine and flow cytometry data. (XLSX)
