# Supplementary figures and images for: Defining characteristics of genital health in South African adolescent girls and young women at high risk for HIV infection
Source: PLoS One. 2019 Apr 4;14(4):e0213975. doi: 10.1371/journal.pone.0213975 (PMC6448899; doi:10.1371/journal.pone.0213975)

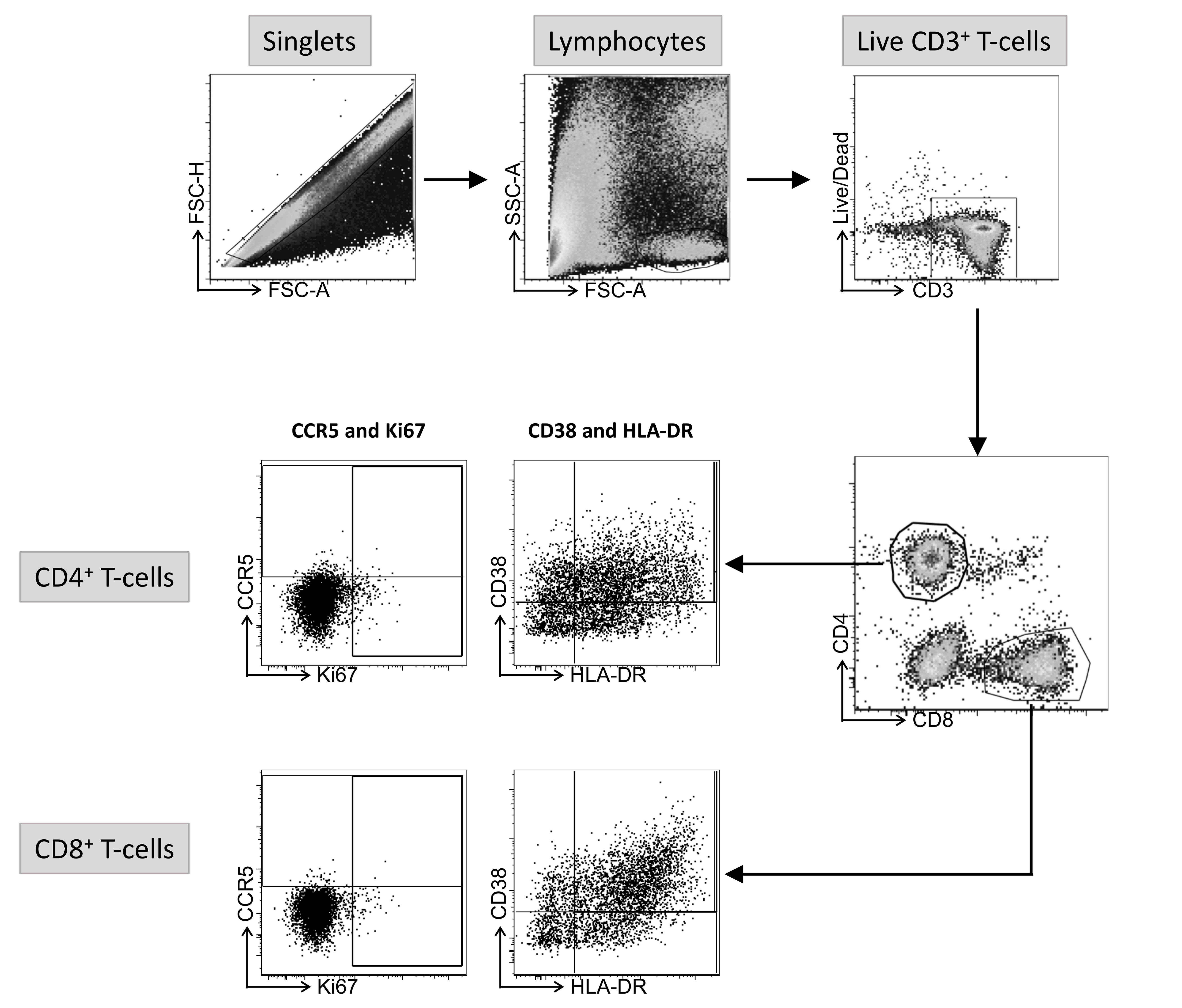

Supplement: S1 Fig — The position of gates for all markers were based on FMOs. (TIFF) [file pone.0213975.s001.tiff]

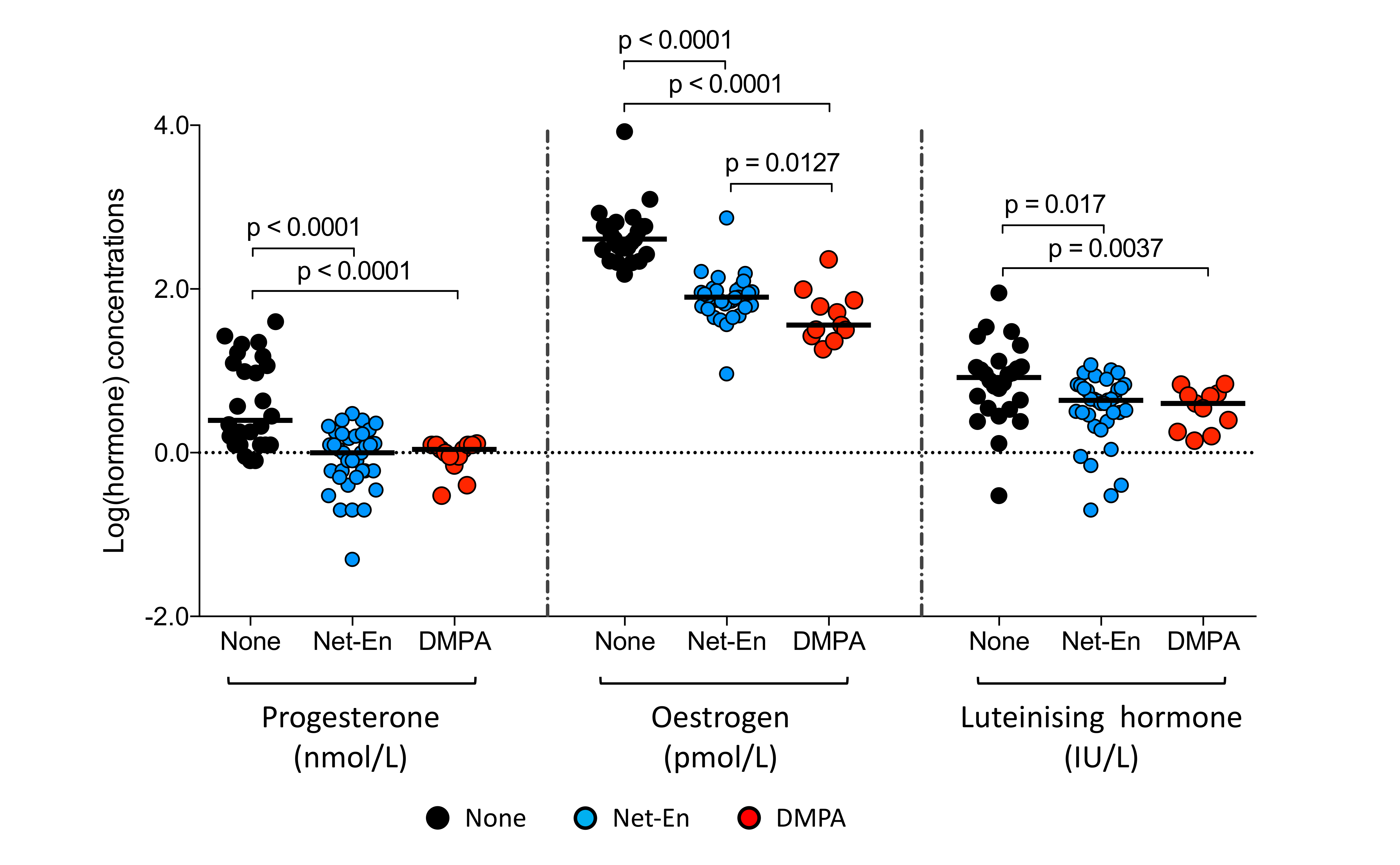

Supplement: S2 Fig — The median concentration of hormone for each group is shown by the solid line. A p value of ≤0.05 was considered significant. (TIF) [file pone.0213975.s002.tif]

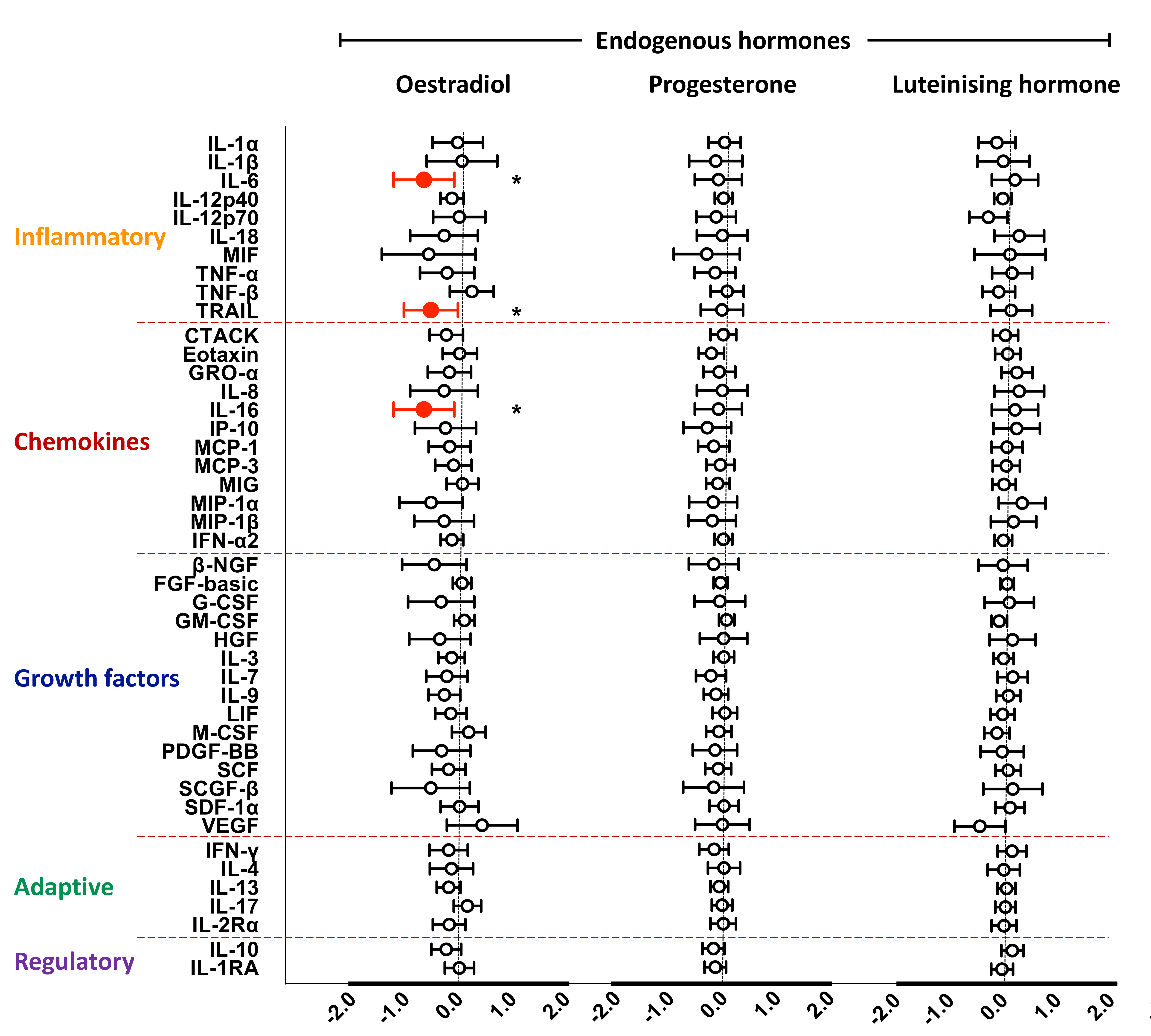

Supplement: S3 Fig — Participant age, hormonal contraceptive use and semen exposure were corrected for. Each association is shown as a β-coefficient and the error bars are the 95% CI. Statistically significant associations are shown in red and p value of ≤0.05 was considered significant. (TIF) [file pone.0213975.s003.tif]

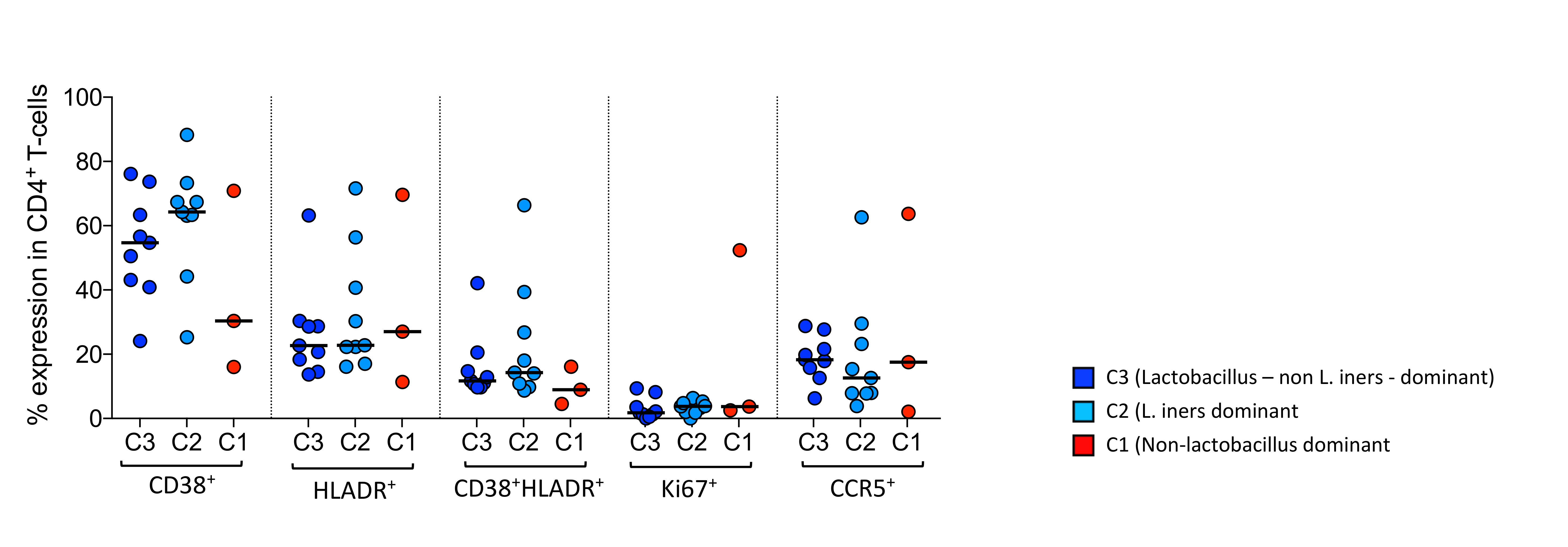

Supplement: S4 Fig — CT1 is shown by red dots, and CT2 and CT3 by light blue and dark blue dots. A p value of ≤0.05 was considered significant. (TIF) [file pone.0213975.s004.tif]

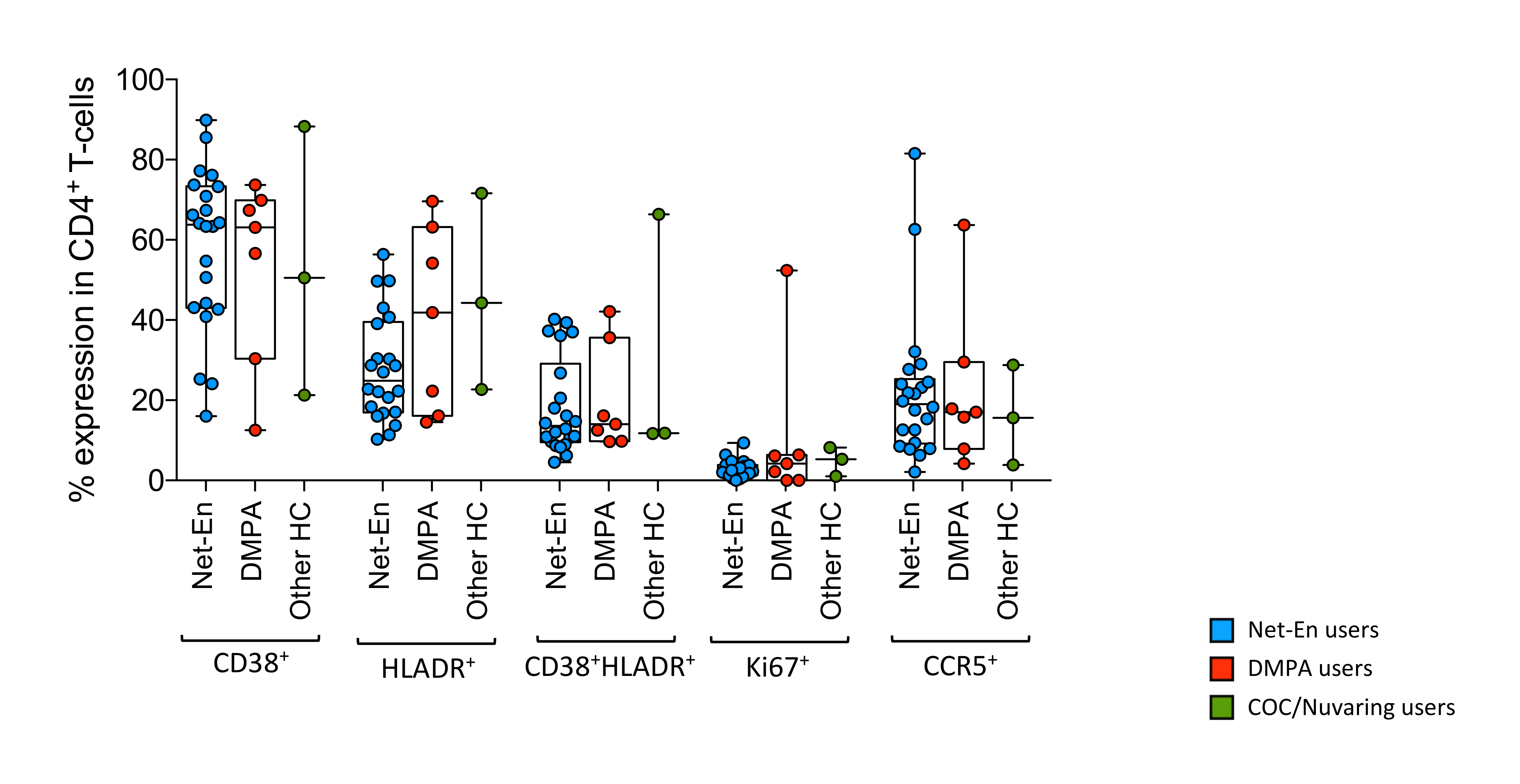

Supplement: S5 Fig — Women using Net-En are shown in blue, women using DMPA in red and those using COC or Nuvaring are shown in green. A p value of ≤0.05 was considered significant. (TIF) [file pone.0213975.s005.tif]
