# Supplementary material for: Defining characteristics of genital health in South African adolescent girls and young women at high risk for HIV infection
Source: PLoS One. 2019 Apr 4;14(4):e0213975. doi: 10.1371/journal.pone.0213975 (PMC6448899; doi:10.1371/journal.pone.0213975)
Supplement: S1 Table — (DOCX) [file pone.0213975.s006.docx]

| **Participant characteristics** | **Variation between women** | | | **Average variation between women** | **Average variation within women over time** |
| --- | --- | --- | --- | --- | --- |
|  | **Visit 1** | **Visit 2** | **Visit 3** |  |  |
| Genital pH | 11.20 | 11.56 | 11.84 | 11.53 | 7.30 |
| CD4+CCR5+ | 124.14 | 128.40 | 124.45 | 125.66 | 64.55 |
| CD4+CD38+ | 49.31 | 47.73 | 37.84 | 44.96 | 33.94 |
| CD4+HLADR+ | 54.37 | 52.36 | 52.21 | 52.98 | 37.77 |
| CD4+CD38+HLADR+ | 64.36 | 58.89 | 59.57 | 60.94 | 54.49 |
| CD4+Ki67+ | 77.16 | 70.15 | 134.35 | 93.89 | 108.71 |
| IL-1α | 8.92 | 9.54 | 11.10 | 9.86 | 7.99 |
| IL-1β | 30.29 | 31.74 | 38.15 | 33.39 | 34.82 |
| IL-6 | 34.14 | 32.16 | 32.16 | 32.82 | 10.37 |
| IL12(p40) | 6.01 | 6.08 | 5.72 | 5.94 | 2.54 |
| IL-18 | 29.61 | 26.08 | 28.80 | 28.16 | 20.88 |
| IL12(p70) | 25.85 | 22.56 | 26.74 | 25.05 | 14.57 |
| MIF | 24.37 | 25.69 | 30.07 | 26.71 | 22.86 |
| TNF-α | 25.35 | 28.83 | 31.73 | 28.64 | 18.23 |
| TNF-β | 21.41 | 20.17 | 19.04 | 20.21 | 7.82 |
| TRAIL | 23.98 | 23.80 | 24.78 | 24.19 | 13.18 |
| β-NGF | 67.73 | 63.31 | 65.70 | 65.58 | 36.89 |
| FGF-basic | 9.98 | 7.01 | 8.03 | 8.34 | 3.02 |
| G-CSF | 24.33 | 24.08 | 29.36 | 25.93 | 21.43 |
| GM-CSF | 4.52 | 4.69 | 4.54 | 4.59 | 1.94 |
| HGF | 22.80 | 22.02 | 26.20 | 23.68 | 12.52 |
| IL-3 | 12.79 | 12.59 | 12.22 | 12.54 | 4.67 |
| IL-7 | 34.40 | 31.25 | 36.41 | 34.02 | 28.21 |
| IL-9 | 25.01 | 23.58 | 23.58 | 24.06 | 9.16 |
| LIF | 19.31 | 18.39 | 18.70 | 18.80 | 7.77 |
| M-CSF | 6.88 | 5.96 | 7.76 | 6.87 | 7.14 |
| PDGF-BB | 58.63 | 27.57 | 41.75 | 42.65 | 84.24 |
| SCF | 19.74 | 19.44 | 18.86 | 19.35 | 7.82 |
| SCGF-β | 31.64 | 31.79 | 31.79 | 31.74 | 17.91 |
| SDF-1α | 23.41 | 24.38 | 24.38 | 24.06 | 9.99 |
| VEGF | 33.85 | 34.28 | 34.41 | 34.18 | 13.09 |
| CTACK | 19.49 | 19.64 | 17.45 | 18.86 | 8.78 |
| Eotaxin | 30.24 | 19.42 | 21.31 | 23.66 | 12.70 |
| GRO-α | 11.94 | 12.03 | 12.51 | 12.16 | 4.64 |
| IL-8 | 17.05 | 17.14 | 18.10 | 17.43 | 12.15 |
| IL-16 | 14.81 | 15.43 | 14.48 | 14.91 | 7.90 |
| IP-10 | 17.70 | 22.96 | 28.76 | 23.14 | 17.61 |
| MCP-1 | 8.77 | 11.42 | 15.16 | 11.78 | 11.57 |
| MCP-3 | 25.51 | 25.74 | 25.74 | 25.66 | 13.83 |
| MIG | 9.86 | 8.22 | 7.98 | 8.68 | 4.80 |
| MIP-1α | 21.79 | 20.46 | 28.68 | 23.64 | 18.06 |
| MIP-1β | 33.45 | 39.82 | 40.91 | 38.06 | 31.08 |
| IFN-α2 | 14.36 | 14.14 | 14.57 | 14.36 | 5.45 |
| IFN-γ | 15.81 | 16.69 | 21.67 | 18.06 | 12.03 |
| IL-4 | 2156.48 | 84.43 | 87.13 | 776.01 | -64.56 |
| IL-13 | 19.85 | 19.85 | 21.05 | 20.25 | 5.41 |
| IL-17 | 20.50 | 16.62 | 16.21 | 17.77 | 6.86 |
| IL-2RA | 18.87 | 18.95 | 18.44 | 18.76 | 6.77 |
| IL-10 | 8.48 | 8.82 | 11.03 | 9.44 | 2.99 |
| IL-1RA | 4.10 | 3.76 | 5.40 | 4.42 | 3.50 |
